# Encapsulated Entomopathogenic Nematodes Can Protect Maize Plants from *Diabrotica balteata* Larvae

**DOI:** 10.3390/insects11010027

**Published:** 2019-12-30

**Authors:** Geoffrey Jaffuel, Ilham Sbaiti, Ted C. J. Turlings

**Affiliations:** FARCE Laboratory, Institute of Biology, Faculty of Sciences, University of Neuchâtel, Rue Emile-Argand 11, 2000 Neuchâtel, Switzerland; ilham.sbaiti@unine.ch (I.S.); ted.turlings@unine.ch (T.C.J.T.)

**Keywords:** entomopathogenic nematodes, encapsulation, *Diabrotica balteata* LeConte, maize, plant protection, biocontrol

## Abstract

To face the environmental problems caused by chemical pesticides, more ecologically friendly alternative pest control strategies are needed. Entomopathogenic nematodes (EPN) have great potential to control soil-dwelling insects that cause critical damage to the roots of cultivated plants. EPN are normally suspended in water and then sprayed on plants or onto the soil, but the inconsistent efficiency of this application method has led to the development of new formulations. Among them is the use of alginate capsules or beads that encapsulate the EPN in favorable conditions for later application. In this study, we evaluated whether alginate beads containing EPN are able to kill larvae of the banded cumber beetle *Diabrotica balteata* LeConte and thereby protect maize plants from damage by these generalist rootworms. EPN formulated in beads were as effective as sprayed EPN at killing *D. balteata*. They were found to protect maize plants from *D. balteata* damage, but only if applied in time. The treatment failed when rootworm attack started a week before the EPN beads were applied. Hence, the well-timed application of EPN-containing alginate beads may be an effective way to control root herbivores.

## 1. Introduction

Environmental and public health concerns linked to the use of chemical insecticides have led to pressures to develop alternative strategies for insect pest control. Biological control using the pests’ natural enemies is commonly recognized as an appropriate alternative [1]. For soil pests, entomopathogenic nematodes (EPN) are excellent biocontrol candidates. EPN from the Steinernematidae and Heterorhabditidae families are obligate parasites of insects [2]. They are associated mutualistically with pathogenic bacteria of the genera *Xenorhabdus* and *Photorhabdus,* respectively, which are lethal to insects [3]. The life cycle of most nematodes includes an egg stage, four juvenile stages, and an adult stage [4]. The only stage that survives outside of a host is the nonfeeding, nondeveloping third stage infective juvenile (IJ), also referred to as dauer juvenile [5]. The IJs search or wait for a suitable insect host and penetrate into the hemocoel by invading through the natural openings (mouth, spiracles, and anus) as well as through some parts of the cuticle. Once inside the body cavity of the insect, the IJs release their symbiont bacteria. As a consequence, the insect dies rapidly, usually within 24–48 h [6,7].

EPN are used against a wide range of insect pests because of their ease of culture, high lethality against key pests, and nonexistent safety issues [8,9]. Nevertheless, the most common formulations for EPN application have a limited shelf life in storage and relatively short survival after application, which are obstacles to the commercialization of EPN-based biopesticides [10,11,12]. To overcome these problems involving EPN storage and application, an increasing number of studies focus on EPN encapsulation in alginate capsules. Alginate is produced by brown algae, and chemically it involves a polymeric linkage network of β-d-mannuronate and α-d-guluronate in varying proportions and sequential arrangements [13]. Kaya and Nelsen (1985) [14] were the first to test the encapsulation of two EPN species, *Heterorhabditis heliothidis* and *Steinernema feltiae*, in calcium alginate by dropping an EPN-containing alginate solution into a CaCl_2_ solution, resulting in capsules containing immobilized EPN. Hiltpold et al. (2012) [15] produced capsules with a liquid core by dripping a mixture of calcium salts of lactic and gluconic acids containing EPN into an alginate solution. Using such capsules containing *Heterorhabditis bacteriophora,* they obtained promising results when they applied the capsules in the field to protect maize plants from damage by the Western corn rootworm *Diabrotica virgifera virgifera* [15]. However, EPN escaped from these capsules within a few days when they were stored at room temperature, which is undesirable for commercialization. EPN escape from alginate capsules was reduced by adjusting the reaction temperature for capsule formation and by applying a post-treatment of alginate capsules with excessive Ca^2+^ [16]. More recently, Kim et al. (2019) [17] adopted a new strategy to limit EPN escape from capsules by adding 18% glycerol, which induces quiescence in EPN. The quiescent EPN are revitalized simply by diluting the glycerol with excessive water. Additionally, alginate capsules are produced by dropping droplets of Ca^2+^ solution containing EPN into the alginate solution, whereas for the formation of alginate beads the process is reversed. This way, more than 4000 EPN can be successfully incorporated in one bead (Kim et al., 2019). Field application of the EPN beads developed by Kim et al. (2019) reduced the damage to maize roots caused by *D. virgifera virgifera* as effectively as EPN that were applied in water.

In the current study, we evaluated the effect of EPN formulated in beads on the larvae of *Diabrotica balteata* LeConte. *D. balteata* larvae feed on a wide range of plants, with preference for plants of the Cucurbitaceae, Rosaceae, Leguminoseae, and Crucifereae families [18]. They damage valuable crops such as squash, cucumber, beet, pea, sweet potato, soybean, bean, okra, corn, lettuce, onion, and various cabbages [18,19]. *Heterorhabditis bacteriophora* was found to naturally parasitize *D. balteata* larvae and to effectively reduce their numbers in pot trials [20], which is why we selected this species to be embedded in the beads for our study. To test the effectiveness of this approach, we carried out two separate laboratory experiments: (1) we evaluated the mortality of *D. balteata* larvae after application of beads with quiescent EPN and compared it to EPN applied in water and (2) we evaluated whether maize plants infested by *D. balteata* larvae benefit from the protection offered by EPN beads.

## 2. Materials and Methods

### 2.1. Nematode Culture and Plant Growth

The *H. bacteriophora* strain was obtained from Andermatt Biocontrol (Grossdietwil, Switzerland) and was maintained using *Galleria mellonella* (L.) larvae as described by Kaya and Stock (1997) [21]. *G. mellonella* larvae were obtained from Au pêcheur SARL (Neuchâtel, Switzerland). The EPN used for the experiments were less than two weeks old.

Maize plants (*Zea mays* L., variety Delprim) were grown from seed in commercial soil (Aussaaterde, RICOTER Erdaufbereitung AG, Aarberg, Switzerland) in individual plastic pots (ø 4 cm, 11 cm high). The plants were grown under natural light conditions (16:8 h L:D) in a greenhouse (24 ± 5 °C) and were watered as needed, they were used for the experiments 8–10 days after planting, when they had four fully expanded true leaves.

### 2.2. EPN Beads Production

Following the method described by Kim et al. (2014), EPN beads were produced by dropping approximately 85–95 μL of the EPN-alginate-glycerol solution (0.5% alginate, 18% glycerol, 0.05% blue dye, 0.075% formaldehyde, and EPN) into a Ca^2+^-glycerol solution (2% CaCl_2_·2H_2_O, 18% glycerol, and 0.075% formaldehyde). Beads were sieved after 20 min polymerization time and quickly rinsed with water. The added glycerol induced the nematodes to enter in a state of quiescence, immobilizing the EPN inside the beads.

### 2.3. Ability of Encapsulated Nematodes to Kill D. balteata Larvae

To evaluate the efficacy of EPN beads containing quiescent *H. bacteriophora* in killing the target insect (*D. balteata* larvae), we conducted an experiment comparing four treatments that were applied to the potted maize plants: (1) EPN-containing beads; (2) EPN suspended in water; (3) empty beads; and (4) control (no application). For the bead treatments, two beads containing approximately 5000 IJs or two empty beads were buried in the soil of the pots (ø 4 cm, 11 cm high) when the maize plants were in the four leaves stage. EPN in water solution were applied onto the soil in 10 mL of water (1000 EPN/mL). Two days after the application of the treatments, ten second instar *D. balteata* larvae were carefully placed on top of the soil. Pots were covered with parafilm to prevent *D. balteata* larvae from escaping. Four days after the larvae were added, larval mortality in the soil of each pot was assessed by counting the retrieved larvae. Each treatment was replicated 20 times in two separate experimental batches. To analyze the data, expressed as percentages, a General Linear Model fitting with a quasipoisson distribution was used. Statistical differences between treatments were calculated using a Tukey test (package “lsmeans”). Statistics were carried in R version 3.4.1 (R Core Team, Vienna, Austria) [22].

### 2.4. Ability of Encapsulated Nematodes to Protect Maize Plants

To evaluate the efficacy of *H. bacteriophora* formulated in EPN beads in protecting plants by killing *D. balteata* larvae, we conducted an additional experiment with bead treatments. In this case, the potted (pot: ø 4 cm, 11 cm high) maize plants were infested with 6 s instar *D. balteata* larvae per pot, and an equal number of plants were left uninfested. Two beads each containing 2000 IJs were applied either (1) the same day when the plants were infested with *D. balteata* larvae (n = 16) or (2) one week after the infestation (n = 12). This time, the beads did not contain glycerol, therefore EPN were not in a quiescent state, which allowed them to escape quicker from the beads and immediately infect the larvae. Pots were covered with parafilm to prevent the larvae from escaping. We measured plant growth, i.e., the difference in plant size (plant height with leaves stretched vertically) at the beginning and the end of the experiment, as well as plant fresh weight one week after applying the *D. balteata* larvae. Two by two comparisons were performed using a Wilcoxon Signed-Rank test performed in R version 3.4.1 [22].

## 3. Results

### 3.1. The Ability of Encapsulated Nematodes to Kill D. balteata larvae

**Figure 1:**
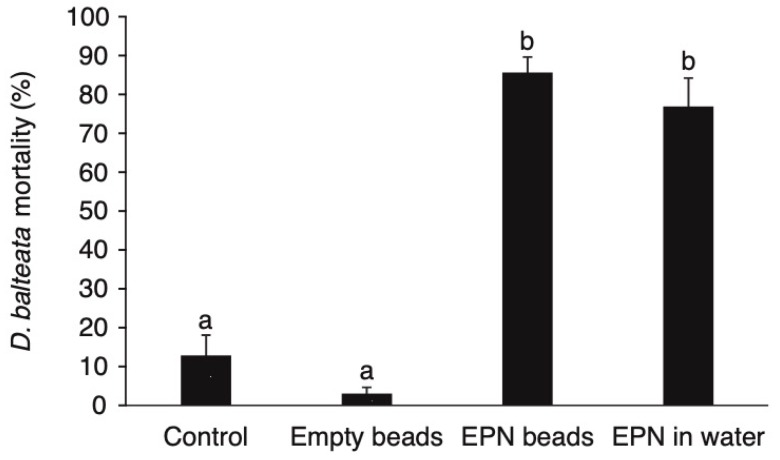
Mortality of *D. balteata* larvae following application of *H. bacteriophora* in aqueous suspension or in alginate beads. Bars represent mean percentage ± SE. Means denoted by different letters are significantly different (*p* < 0.05).

*H. bacteriophora* applied in alginate beads (EPN beads) and in aqueous suspension (EPN in water) were equally effective in killing *D. balteata* larvae. Overall, the differences among the four treatments were highly significant (DF = 63, F = 41.09, *p* < 0.001) but the mortality caused by EPN beads (86%) did not significantly differ from mortality caused by free EPN (77%) (*p* = 0.2). Empty beads had no effect on larval mortality (*p* = 0.15) (Figure 1).

### 3.2. The Ability of Encapsulated Nematodes to Protect Infested Maize Plants

**Figure 2:**
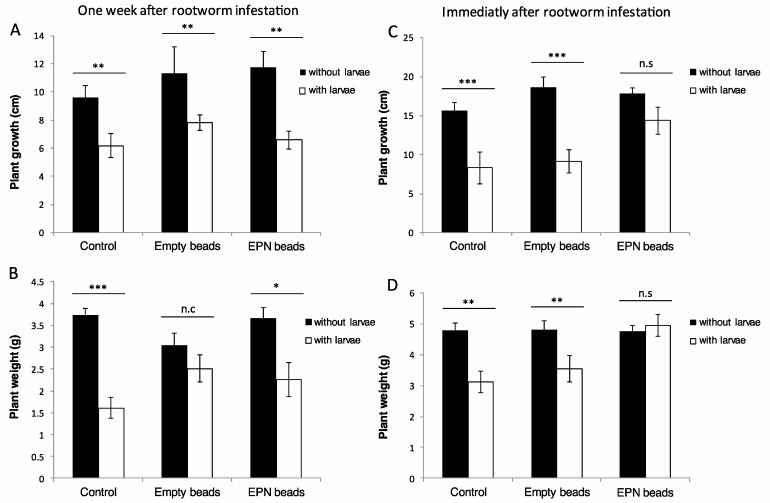
Increase in size (**A**,**C**) and fresh weight (**B**,**D**) (mean ± SE) of maize plants infested or not infested with *Diabrotica balteata* larvae, after application EPN-containing or empty beads. The application was either immediately after larval infestation (**A**,**B**) or one week after larval infestation (**C**,**D**). Probability levels: * *p* < 0.05, ** *p* < 0.01, *** *p* < 0.001, n.s., non-significant.

*H. bacteriophora* applied in alginate beads a week after infestation failed to protect the plant from *D. balteata* damage. In this trial, plant growth and weight were significantly reduced by *D. balteata* in all treatments except for the plant weight in the empty beads treatment (for growth; control: W = 31, *p* = 0.01, empty beads: W = 30, *p* = 0.01, EPN beads: W = 35, *p* = 0.03, and for weight; control: W = 13, *p* ≤ 0.001, empty beads: W = 60, *p* = 0.5, EPN beads: W = 28, *p* = 0.01) (Figure 2A,B).

When EPN beads were applied at the same time that the plants were infested, the EPN were able to reduce root damage in a way that enhanced plant performance. In this case, there was again a strong negative effect of *D. balteata* on plant growth and weight in the control and empty bead treatments (for growth; control: W = 35, *p* ≤ 0.001, empty beads: W = 23.5, *p* < 0.001, and for weight; control: W = 48.5, *p* = 0.005, empty beads: W = 56.5, *p* = 0.007) but the EPN bead treatment completely neutralized the negative effect of the larvae on plant growth and weight (EPN beads: W = 95, *p* = 0.9 and for growth; EPN beads: W = 63, *p* = 0.1) (Figure 2C,D).

## 4. Discussion

Studies that tested EPN to control *Diabrotica* spp. have shown inconsistent results, ranging from low to very high levels of efficacy [23]. EPN can cause high levels of mortality in *Diabrotica* spp. laboratory tests, but the success of field applications depends largely on environmental conditions, formulation type, and application techniques [23,24,25]. Several recent research projects focused on encapsulation of EPN in Ca^2+^-alginate capsules to protect them from the abiotic constrains [14,15,16,17,26]. In our study, we tested the potential of encapsulated quiescent *H. bacteriophora* in beads, described by Kim et al. [16], to kill the larvae *D. balteata* under laboratory conditions. During the production of the beads, quiescence was induced by adding 18% glycerol. After application of the beads into sufficiently moist soil, quiescence was broken and the EPN readily escaped from the alginate beads and infected the target insect. Under laboratory conditions, the control (larval mortality) provided by the beads containing quiescent EPN was just as good as applying equal numbers of EPN in aqueous suspension (Figure 1). Thus, the advantages of encapsulating EPN mainly consist of prolonged shelf life, a slow and controlled release, and easy and compact storage [14,15,16,17,26,27].

We also confirmed that the bead application reduced *D. balteata*-inflicted root damage to an extent that it enhanced plant performance. The timing of the bead application was found to be critical to obtain a protective effect of EPN on maize plants. Indeed, when the *D. balteata* infestation was initiated one week before bead application, the EPN were not able to prevent a significant reduction in either plant growth or weight (Figure 2A,C). However, when EPN beads were applied at the same time that the plants were infested, the EPN provided sufficient protection to the plant and reduced the damage to a level that it had no effect on plant performance (Figure 2B,D). The difference observed between the two applications schedules can be explained by two factors: (1) the roots were already heavily damaged one week after infestation and the application of EPN was too late to reduce this damage and (2) the EPN were not able to reach the *D. balteata* larvae because they had tunneled into the root system. This important effect of timing should also be taken into consideration if we wish to use of glycerol to trigger EPN quiescence. Soil moisture and other factors will play a role in the recovery process from quiescence. It is essential that this recovery occurs before the pest larvae start damaging the roots. More studies on the effect of glycerol on EPN release from the beads in different soils will be needed to be able to pinpoint the ideal timing of application to achieve optimal plant protection. Overall, the results of these experiments confirm the potential of EPN formulated in beads to kill rootworms and to protect crops from root damage.

## 5. Conclusions

The alginate beads containing entomopathogenic nematodes were as efficient as entomopathogenic nematodes applied in water to kill larvae of the banded cucumber beetle, *Diabrotica balteata,* and were able to protect maize plants from larval damage under laboratory conditions. The timing of alginate beads application was critical to ensure an effective protection of the plants.
